# Screening of important metabolites and KRAS genotypes in colon cancer using secondary ion mass spectrometry

**DOI:** 10.1002/btm2.10200

**Published:** 2020-11-17

**Authors:** Kookrae Cho, Eun‐Sook Choi, Sung Young Lee, Jung‐Hee Kim, Dae Won Moon, Jong‐Wuk Son, Eunjoo Kim

**Affiliations:** ^1^ Division of Electronic Information System Research Daegu Gyeongbuk Institute of Science and Technology (DGIST) Daegu Republic of Korea; ^2^ Division of Bio‐Fusion Research Daegu Gyeongbuk Institute of Science and Technology (DGIST) Daegu Republic of Korea; ^3^ Division of Technology Business, National Institute for Nanomaterials Technology (NINT) Pohang University of Science and Technology (POSTECH) Pohang Republic of Korea; ^4^ Department of New Biology Daegu Gyeongbuk Institute of Science and Technology (DGIST) Daegu Republic of Korea

**Keywords:** biomarker screening, colorectal cancer, KRAS somatic mutation, support vector machine learning algorithm, time‐of‐flight secondary ion mass spectrometry (TOF‐SIMS)

## Abstract

Time‐of‐flight secondary ion mass spectrometry (TOF‐SIMS) is an imaging‐based analytical technique that can characterize the surfaces of biomaterials. We used TOF‐SIMS to identify important metabolites and oncogenic KRAS mutation expressed in human colorectal cancer (CRC). We obtained 540 TOF‐SIMS spectra from 180 tissue samples by scanning cryo‐sections and selected discriminatory molecules using the support vector machine (SVM) algorithm. Each TOF‐SIMS spectrum contained nearly 860,000 ion profiles and hundreds of spectra were analyzed; therefore, reducing the dimensionality of the original data was necessary. We performed principal component analysis after preprocessing the spectral data, and the principal components (20) of each spectrum were used as the inputs of the SVM algorithm using the R package. The performance of the algorithm was evaluated using the receiver operating characteristic (ROC) area under the curve (AUC) (0.9297). Spectral peaks (*m/z*) corresponding to discriminatory molecules used to classify normal and tumor samples were selected according to *p*‐value and were assigned to arginine, α‐tocopherol, and fragments of glycerophosphocholine. Pathway analysis using these discriminatory molecules showed that they were involved in gastrointestinal disease and organismal abnormalities. In addition, spectra were classified according to the expression of KRAS somatic mutation, with 0.9921 AUC. Taken together, TOF‐SIMS efficiently and simultaneously screened metabolite biomarkers and performed KRAS genotyping. In addition, a machine learning algorithm was provided as a diagnostic tool applied to spectral data acquired from clinical samples prepared as frozen tissue slides, which are commonly used in a variety of biomedical tests.

## 1 INTRODUCTION

Certain lipids and metabolites are closely related to the initiation and progression of cancers. Metabolomic analysis to diagnose cancer and assist therapeutic treatments using mass spectrometry has been widely studied for various tumor types.^1^, ^2^ In conventional mass spectrometric analyses of metabolites, extraction of the aqueous or fat‐soluble fraction from tissues should be performed first, and subsequently the extracted fraction can be analyzed by gas (GC‐MS) or liquid (LC–MS) chromatography–mass spectrometry.^3^, ^4^

Time‐of‐flight secondary ion mass spectrometry (TOF‐SIMS) has been used to analyze surface chemical compositions of samples, via identification of positive and negative secondary ions, with high resolution and sensitivity.^5^, ^6^ Recently, TOF‐SIMS has been used to study complex biological molecules on the surfaces of tissue specimens prepared by cryosectioning.^7^, ^8^ In TOF‐SIMS studies of cancer, comparative analyses of the surface images in diseased and normal tissues revealed discriminatory images of disease states, supported by known molecular markers.^7^, ^9^ These studies indicate that TOF‐SIMS is a promising technology to analyze small lipids and metabolites without the separation of lipid or aqueous fractions from the samples.

Current state‐of‐the‐art TOF‐SIMS data sets consist of large amounts of raw data for a single experiment, ranging from gigabytes to terabytes in size, and typically containing 10^4^–10^6^ *m/z* data. Therefore, computer memory shortages and long processing times when using standard desktop computers are likely for the application of classification techniques based on software designed for traditional statistical methods such as principal components analysis (PCA), partial least square‐discriminant analysis (PLS‐DA), and orthogonal partial least squares discriminant analysis (OPLS‐DA).^10^ A common method to deal with these issues is to perform a feature selection step such as peak‐picking before data analysis. This reduces the number of variables and the data set size by eliminating spectral bins that do not contain peak centers. Other methods used to reduce the size of the data include spectral binning, which involves averaging ion intensities over multiple *m/z* bins. Several studies have been reported on the analysis of TOF‐SIMS data using the abovementioned methods, including feature selection, in which ten to thirty samples were used to verify spectral variations.^11^, ^12^, ^13^, ^14^

In fact, several groups have investigated efficient machine learning algorithms and techniques capable of handling the large amounts of data from TOF‐SIMS measurements for imaging‐based mass spectrometric or chemical analyses.^10^, ^15^ However, studies on computational methods that can identify disease states by extraction of clinically important molecular information from TOF‐SIMS data sets are insufficient. It requires minimal loss of information and reduced dimensionality. Therefore, optimized methods should be developed to support TOF‐SIMS, to obtain essential information on diseases from clinical samples.

In this study, we applied TOF‐SIMS analysis to the screening of tumor‐related metabolites in colorectal cancer (CRC), which is the third most deadly and fourth most common cancer in the world.^16^ CRC is a malignant organ tumor and is common among elderly people. Research on CRC has been extensive, but few studies have reported the application of TOF‐SIMS to the metabolomic analysis of CRC tissues. In one study, 48 amino acids peak from TOF‐SIMS measurements were analyzed by PCA to distinguish cancerous areas from normal colon mucosa with the aim of developing a new cancer diagnostic technique.^17^ In our study, a support vector machine (SVM) algorithm was applied in the analysis of mass profiles, to improve the analysis method and allow the extraction of more detailed information, including important biomarkers, from TOF‐SIMS data sets. SVM is a supervised model for linear regression analysis on nonlinear data sets. SVM‐based classification of samples in the analysis of metabolite profiles from GC–MS and LC–MS has been reported,^18^, ^19^ but efficient SVM models optimized for TOF‐SIMS data analysis using clinical samples have not yet been defined.

We prepared specimens from CRC patients and analyzed each one using TOF‐SIMS to identify lipid and metabolite biomarkers that are enhanced in tumor tissues. The SVM algorithm was optimized for the classification of the spectra, and important molecules were selected as CRC‐specific biomarkers. The feasibility of the biomarkers was evaluated by comparing them with those selected by conventional GC–MS analysis. Furthermore, we propose that the mass profile from TOF‐SIMS can discriminate KRAS (Kirsten rat sarcoma 2 viral oncogene homolog) somatic mutation genotypes, of which there is a high incidence rate in CRC, >40%.^20^

## 2 RESULTS AND DISCUSSION

### 2.1 Data acquisition by TOF‐SIMS

**FIGURE 1:**
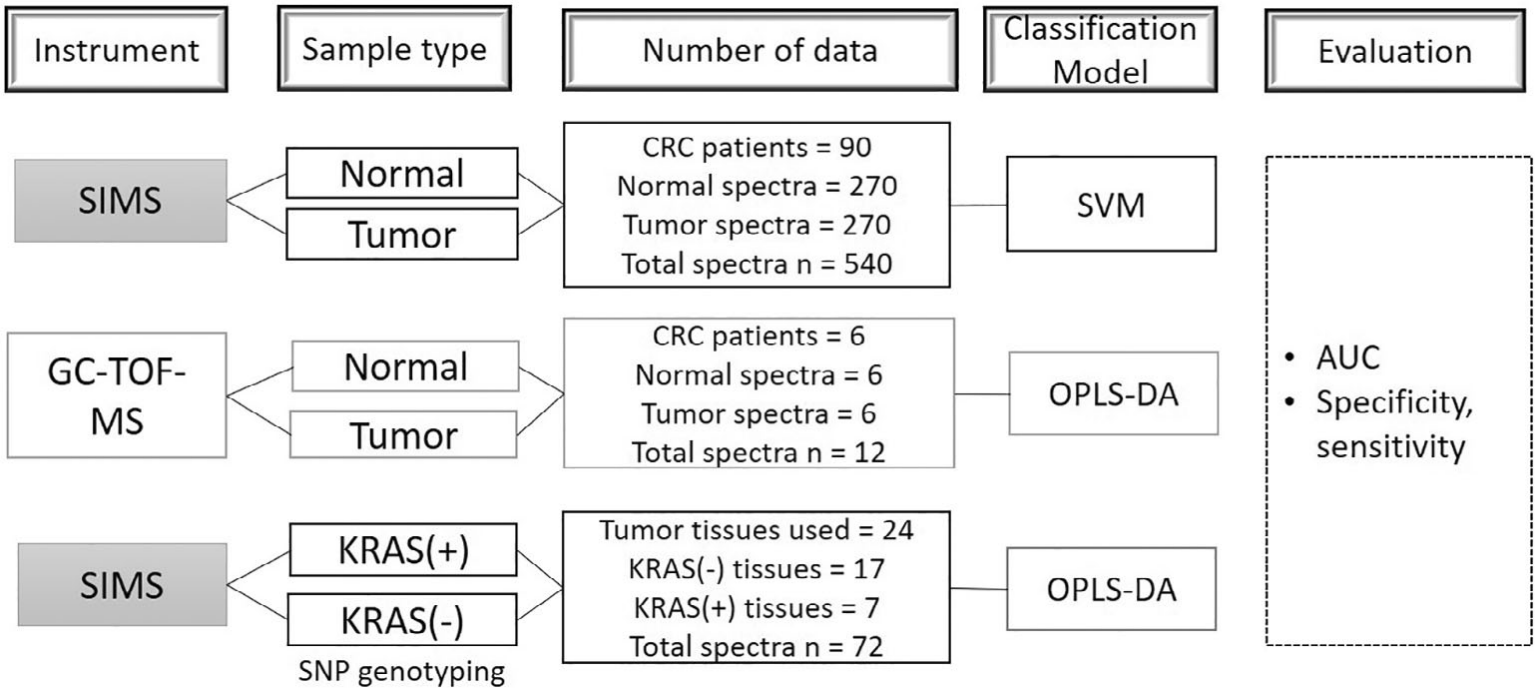
Schematic diagram of analysis methods and steps used in this study

The experimental design and samples used in this study are summarized in Figure 1. To increase the reliability of the machine learning algorithm, we collected a greater number of CRC patient samples than is usual for a metabolomic analysis: a total of 180 specimens from 90 CRC patients were collected. Figure S1A shows a representative tissue image analyzed by TOF‐SIMS. The acquired data were the intensity profiles of each ion released from the tissue surface, which were in the range of 100 to 1300 *m*/*z*. Over 859,000 ions were detected from samples with areas of 250 μm × 250 μm. The image on the left in Figure S1A shows a tumor tissue stained with hematoxylin and eosin (H&E), the optical image of the specimen to be measured by TOF‐SIMS is shown in the middle, and the image on the right displays the data acquired from the spatial distribution of the ion signals at each pixel (256 × 256 pixels). Figure S1B shows representative mass spectra of normal and tumor tissue from a single patient. Most of the ion masses were detected at 100–350 *m*/*z*, as shown in the figure.

### 2.2 Optimization and classification accuracy of the SVM model for the analysis of TOF‐SIMS data

**FIGURE 2:**
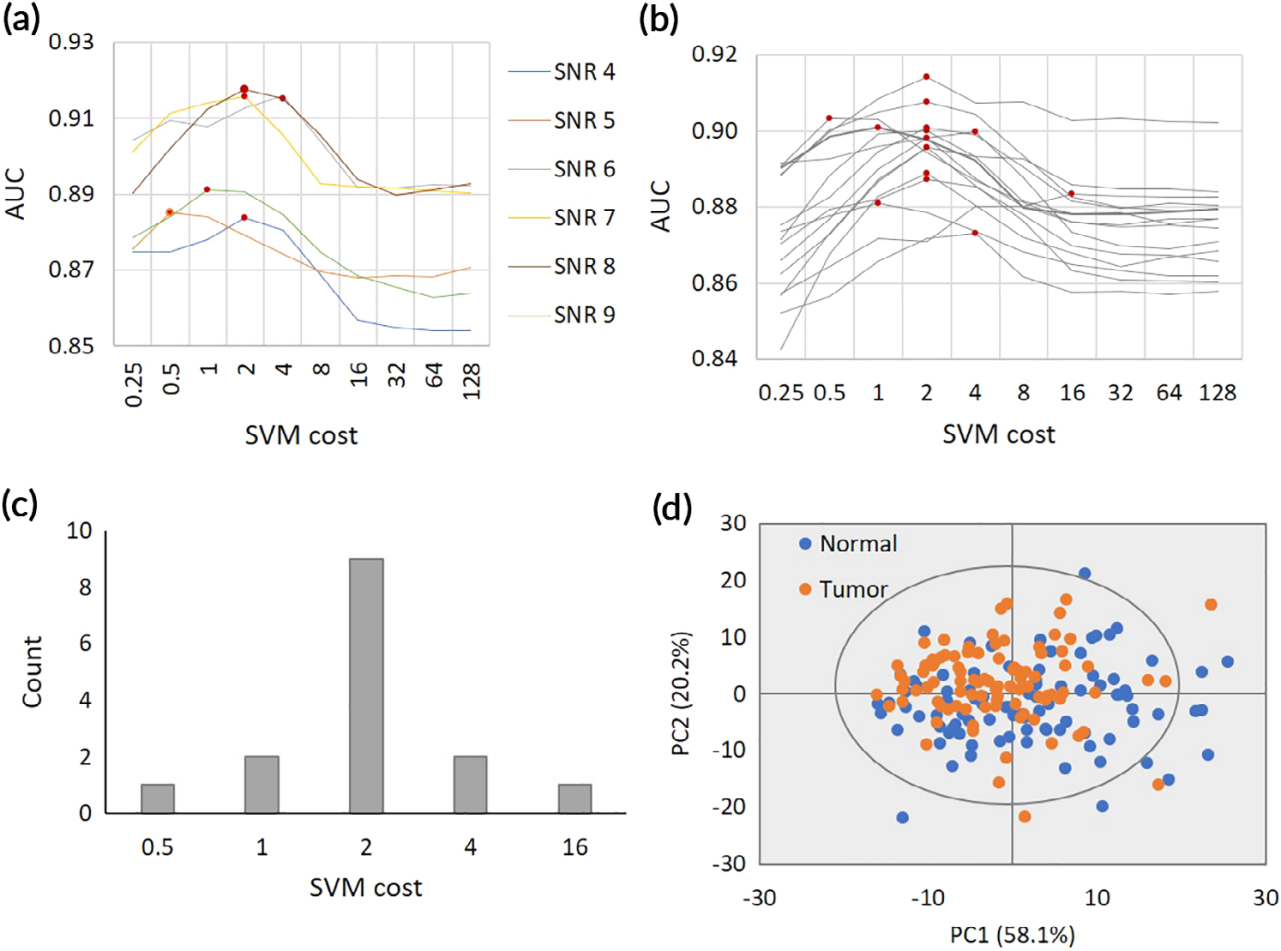
Preprocessing and optimization of SVM algorithm. (a) Determination of optimized SNR based on the AUC value. (b) Optimization of SVM score by cross validation. (c) Number of models with the highest AUC values according to SVM cost. (d) PCA score plot for normal and tumor tissue spectra

Data preprocessing was performed using the MALDIquant package, and the parameters used are listed in Table S1. To optimize the preprocess parameters, the selected signal‐to‐noise ratio (SNR) was varied from four to nine during peak detection. Figure 2a shows that the highest area under the curve (AUC) value using a receiver operating characteristic (ROC) curve occurred when the SNR for peak detection was set to eight.

Cross‐validation was performed for tuning the cost parameter, and Figure S2 shows a schematic diagram of the cross‐validation process for the SVM model development.^21^ Figure 2b indicates that 8 among 14 models produced by the cross‐validation process had the highest AUC values, for which the SVM cost was two. Figure 2c shows the number of models that were evaluated with the highest AUC when the SVM cost was 0.5, 1, 2, 4, and 16. As a result, the SVM model was optimized by setting the SNR to eight and the SVM cost to two.

PCA analysis was applied to the preprocessed data sets of normal and tumor samples to investigate the structure of the mass profiles as variables for unsupervised classification (Figure 2d). Twenty principal components were generated, and the first two components captured 78.3% (58.1% and 20.2%) of the total variance in the data. The PCA loadings were not clearly separated between the normal and tumor groups, indicating that the data structure acquired from TOF‐SIMS analysis was extremely complicated. In this case, OPLS‐DA could be used as an alternative method because PCA failed to separate the groups. However, based on a study using data that had been incompletely separated into groups by PCA, it was reported that OPLS‐DA could easily yield statistically unreliable group separation.^22^

In this study, an SVM model was used to analyze TOF‐SIMS spectra using R packages. We used 20 principal components of each observation as input features for the SVM algorithm to reduce the dimensionality of the TOF‐SIMS spectra. The combined application of PCA and SVM was reported in previous studies for the classification of groups with high dimensionality.^23^, ^24^

The prepared SVM model was evaluated for its ability to classify the spectra from tumor and normal tissues based on several parameters, including a ROC curve. Table S2 provides a summary of the evaluation results. The sensitivity and specificity of the prediction model were 0.8387 and 0.8817, respectively, and the accuracy was 0.8602. The AUC was 0.9207. Fleiss' kappa statistic for the SVM algorithm was 0.7024; a higher kappa value indicates stronger agreement, and a kappa value of one indicates perfect agreement. Based on Landis' categories,^25^ the kappa value for the SVM in this study was interpreted to indicate “substantial agreement (kappa range, 0.61–0.80)”. These results indicated that the optimized SVM algorithm was able to classify normal and tumor tissues from CRC patients with substantial accuracy.

There are several reports on the use of SVM models for mass spectrometric analysis. Anderson et al., reported an SVM classification model to identify proteins from peptide profiles produced by a tryptic digest in proteomic analysis.^26^ They used 696 peptide mass values cleaved from 47 proteins, and the AUC was determined to be 0.920–0.988. In another case of the application of SVM to mass spectrometric analysis by Guan et al., serum mass data produced by LC–MS was used to classify ovarian cancer samples.^27^ In this case, the accuracy of the models was determined to be 0.917–0.972, using 72 patient samples and hundreds of mass values for each patient. Because this study was focused on recognizing panels of important features for the classification of ovarian cancer serum, its purpose was the same as that of the present study, even though the target cancer type was different. However, the different instrument used produced different data sets, and different data preprocessing was used. These differences necessitated the customization of the model development for our study.

In the studies by Anderson et al. and Guan et al., high AUC and accuracy values over 0.9 were achieved, which implies that the application of SVM algorithms to the classification of biological data is a promising approach. In our study, the AUC obtained for the classification of colon cancer tissues (0.9207) was comparable to those reported for other studies. Moreover, our study involved the processing and analysis of very large data sets composed of 859,000 mass values from each specimen. Even though we could not compare the prediction performance of the model with those of other TOF‐SIMS studies due to the unavailability of the previous data, we can conclude that the constructed SVM algorithm could be a possible model for the analysis of TOF‐SIMS data, for example, for the selection of important molecules and for disease diagnosis using clinical samples.

### 2.3 Discriminatory metabolites for normal and tumor tissues selected from TOF‐SIMS spectra

Within clinical settings, one of the most important considerations for a machine learning model is the identification of important molecules that can be used to discriminate between normal and tumor tissues.^28^ In this study, we selected discriminatory metabolites based on the *p*‐values generated by the SVM algorithm.

Table 1 shows the 20 features selected as the top variables, and these variables could be grouped in some cases; the peak *m*/*z* value showing the highest intensity within a group was finally selected as the discriminatory metabolite in these cases. The peaks were assigned to arginine, tocopherol, and fragments of glycerophosphocholines (G‐PCs) of different sizes, based on previous TOF‐SIMS analysis results.^29^, ^30^, ^31^ In fact, four of the seven metabolites were related to G‐PCs, which have been observed widely in tissues analyzed by TOF‐SIMS.^32^ These are usually detected in fragmented forms in lipid‐related metabolic pathways in mammalian systems, and can be identified using ^15N^phosphocholine molecules.^31^ Lipid analyses of samples from cancer tissue displayed an increase in phospholipid content as compared to non‐cancerous adjacent healthy tissue.^33^ For example, concentrations of the two major phospholipid components, phosphatidylcholine and phosphatidylethanolamine, were found to increase with breast‐cancer tumor grade, indicating that phospholipid synthesis was dependent on tumor progression.^34^

**TABLE 1:** Discriminatory molecules for normal and tumor tissues generated by SVM analysis of TOF‐SIMS data

Top variables	Peak value (*m*/*z*)	*p*‐value	Fold change (CRC/Normal)	Assignment	Formula	Reference
102.08	**102.08**	6.57 × 10^−11^	1.33	Arginine	C_4_H_12_N_3_^+^	[27]
104.11, 104.36 104.38, 104.42 104.44, 104.46 104.47, 104.51 104.63	**104.11**	5.82 × 10^−7^	1.29	G‐PC/SM	C_5_H_14_NO^+^	[28, 29]
150.06	**150.06**	1.10 × 10^−9^	1.35	α‐tocopherol	C_10_H_14_O^+^	[27]
166.06	**166.06**	7.35 × 10^−7^	1.25	G‐PC	C_5_H_13_NPO_3_^+^	[28]
184.74, 184.78 184.81, 184.84 184.90, 184.92	**184.81**	1.59 × 10^−6^	1.25	G‐PC/SM	C_5_H_15_NPO_4_^+^	[28, 29]
224.10	**224.11**	2.33 × 10^−7^	1.38	G‐PC	C_7_H_15_NPO_5_^+^	[28, 29]
238.12	**238.12**	2.37 × 10^−7^	1.38	G‐PC	C_8_H_17_NPO_5_^+^	[28]

Abbreviations: G‐PC, Glycerophosphocholine; SM, Sphingomyelin.

**FIGURE 3:**
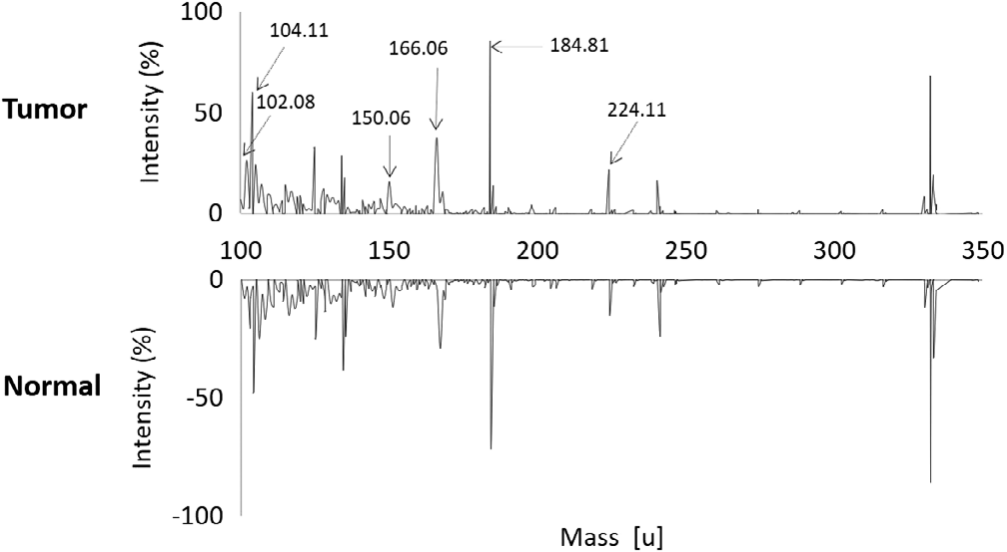
Performance of classification using the SVM algorithm. Comparison of TOF‐SIMS spectra averaged over 270 measurements. Arrows indicate the peaks of the discriminatory molecules determined by the SVM model. Numbers are m/z values for each peak

Figure 3 shows the averaged spectra of normal and tumor tissues, acquired by TOF‐SIMS measurement. The selected discriminatory metabolites, *m*/*z* = 102.08, 104.11, 150.06, 166.06, 184.81, and 224.11, are observed in the main peaks of the overall tumor spectrum. Interestingly, compared with the normal tissue, all of these were upregulated in the tumor tissue, and the fold changes in tumor tissues are also listed in Table 1. In fact, the tumor specimens used in this study were not informed on their types and stages of CRC, but we used tumor samples with an information that the tumor ratios were over 70%. Therefore, these discriminatory molecules are proposed as common biomarkers that can be applied to the diagnosis of all types of CRC, if TOF‐SIMS is used for clinical diagnosis.

### 2.4 Discriminatory metabolites from GC–TOF‐MS spectra

To carry out a feasibility comparison between the potential biomarkers selected by TOF‐SIMS analysis and those selected by another method, gas chromatography–time‐of‐flight mass spectrometry (GC–TOF‐MS) analysis was performed on additional CRC patient tissue samples. The sample number used in GC–TOF‐MS analysis was 12 (six normal tissue and six tumor tissue samples). Pretreatment of samples using methanol extraction was performed to separate lipids and small molecule fractions for gas chromatography. By GC–TOF‐MS analysis, 14,600 mass data points per metabolite profile coupled with retention time were acquired from each sample. The quantity of data produced by GC–TOF‐MS means that the results can be analyzed using OPLS‐DA (SIMCA+ software), as reported previously.^35^, ^36^

**FIGURE 4:**
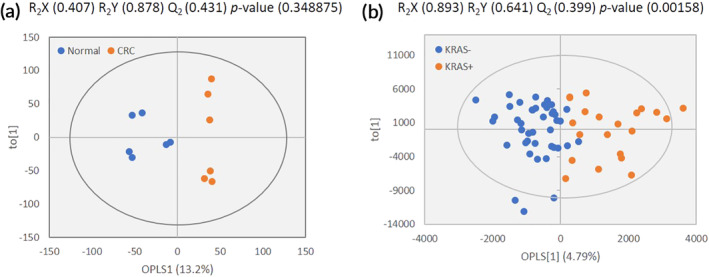
OPLS‐DA score plots for the classification of tumor and KRAS(+) tissues. (a) Score plot for classification of normal and tumor tissues. (b) Score plot for classification of KRAS(+) and KRAS(−)

The OPLS‐DA score plot is shown in Figure 4a; the separation of the samples in the score plot is apparent, and the AUC and accuracy are determined to be 1. The resulting performance statistics were *R* ^2^ *X* = 0.407, *R* ^2^ *Y* = 0.878, and *Q* ^2^ = 0.431 (Table S3); thus, the *R* ^2^ *X* and *Q* ^2^ values did not meet the criteria of a good prediction model (*R* ^2^ *X*, *R* ^2^ *Y*, and *Q* ^2^ > 0.5). Table 2 shows the discriminatory molecules identified by GC–TOF‐MS analysis. The criteria for the selection of these molecules were that their variable importance parameter (VIP) was greater than one. Thirteen molecules were selected as discriminatory molecules; however, the *p*‐values of those molecules did not meet the criteria for statistical significance (*p <* 0.05).

**TABLE 2:** Discriminatory molecules in tumor tissues analyzed by GC–TOF‐MS

Discriminatory molecules	RT (min)	Mass	VIP	*p*‐value
Threonic acid	9.62	292	1.751	0.055
*myo*‐Inositiol	13.41	191	1.578	0.091
Ethanolamine	6.98	174	1.492	0.113
Pantothenic acid (vitamin B5)	12.72	103	1.342	0.160
Glucose	12.16	205	1.329	0.164
Glycerol	7.07	218	1.302	0.174
Hydroxylamine	5.43	133	1.273	0.185
*N*‐Acetyl‐D‐glucosamine	13.35	202	1.228	0.202
Hypoxanthine	11.62	265	1.212	0.209
Oxalic acid	5.64	219	1.066	0.273
Lactic acid	4.89	117	1.058	0.277
Stearic acid	14.12	117	1.047	0.282
Uridine monophosphate	17.40	169	1.033	0.289

Abbreviation: RT, retention time.

The number of samples used in this study was relatively lower than those used in previous studies on CRC biomarkers using GC–TOF‐MS. Phua et al.^37^ and Qui et al.^38^ used 21 and 376 total patient tissue samples, respectively. However, the reported OPLS‐DA performance statistics were quite comparable to those of our study: for Phua et al., *R* ^2^ *X* = 0.407; *R* ^2^ *Y* = 0.996; *Q* ^2^ = 0.35, and for Qui et al., *R* ^2^ *X* = 0.374; *R* ^2^ *Y* = 0.706; *Q* ^2^ = 0.532. In addition, several molecules listed in Table 2—*myo*‐inositol, lactic acid, hypoxanthine, glycerol, uracil, and glucose—were also selected as discriminated molecules in the study of Qiu et al. However, the panels of proposed biomarkers in these literature studies were substantially different from each other as well as from that of the current study. This result indicates that the CRC‐specific metabolites selected by GC–TOF‐MS are not generally reproduced by different studies, irrespective of the sample numbers. The multiple lipid‐extraction steps using organic solvents might have contributed to the different results from the GC–TOF‐MS measurements.

### 2.5 Comparison of TOF‐SIMS and GC–TOF‐MS biomarkers by pathway analysis

Pathway analysis can demonstrate important diseases and biological functions related to discriminatory molecules. The top pathways involved for the important molecules selected by TOF‐SIMS and GC–TOF‐MS are listed in Table 3. Gastrointestinal disease, organismal injury and abnormality, and endocrine system disorders were identified as the top three pathways related to arginine, tocopherol, and G‐PCs. Developmental disorders, hereditary disorders, and metabolic diseases were proposed to be related to glucose, hypoxanthine, stearic acid, and glycerol, which were selected as discriminants by GC–TOF‐MS analysis. The top pathways proposed by TOF‐SIMS analysis included gastrointestinal diseases, which could be closely related to CRC. This result indicated that the discriminatory molecules selected by TOF‐SIMS analysis had enhanced feasibility, with respect to those selected by GC–TOF‐MS analysis, as CRC‐specific metabolite biomarkers. Therefore, TOF‐SIMS measurements can be considered a potential screening method to identify important molecules released from the surface of tumor tissues as specific signals. In addition, the SVM algorithm optimized for the classification of tumor tissues efficiently analyzed the TOF‐SIMS data, deriving discriminatory molecules with high significances.

**TABLE 3:** Pathways related to the discriminatory molecules determined by TOF‐SIMS and GC–TOF‐MS analyses

	Diseases and bio function	*p*‐value	Related molecules
TOF‐SIMS	Gastrointestinal disease	4.16 × 10^−2^–5.73 × 10^−4^	arginine, tocopherol, G‐PC
	Organismal injury and abnormality	4.15 × 10^−2^–5.73 × 10^−4^	arginine, tocopherol, G‐PC
	Endocrine system disorder	1.83 × 10^−2^–7.37 × 10^−4^	arginine
GC–TOF‐MS	Developmental disorder	7.53 × 10^−3^–6.06 × 10^−5^	Glucose, hypoxanthine, stearic acid, glycerol
	Hereditary disorder	7.35 × 10^−3^–6.06 × 10^−5^	Glucose, hypoxanthine, stearic acid
	Metabolic disease	1.61 × 10^−2^–6.06 × 10^−5^	Glucose, hypoxanthine, stearic acid

### 2.6 Prediction of KRAS mutations in CRC tissues

The abnormal activation of KRAS plays a critical role in tumor initiation and progression with the highest frequency of human malignancies.^39^, ^40^ Treatment options for CRC patients harboring a KRAS mutation are limited and their mortality rate is high;^41^ therefore, it is crucial to determine whether KRAS is mutated in the tumor tissues of CRC patients. If it were possible to perform KRAS genotyping with simultaneous diagnosis of CRC, it would help in the establishment of more efficient therapeutic programs for CRC patients. In recent years, KRAS mutation assays become important companion diagnostic tests.^42^

In this study, the KRAS genotype was determined by analyzing the TOF‐SIMS spectra. Out of the 90 patient tumor samples used for SIMS measurement, 24 samples were randomly selected, and single nucleotide polymorphism (SNP) genotyping for KRAS mutations G12D, G12A, and G12V was performed by nucleotide sequencing. Then, metabolite profiles were analyzed to classify them according to expression of the KRAS(+) and KRAS(−) mutations.

As a result, we were able to classify KRAS(+) and KRAS(−) tissues by analyzing their TOF‐SIMS spectra. As shown in Figure 4b, the performance statistics were *R* ^2^ *X* = 0.893, *Q* ^2^ = 0.399, and *R* ^2^ *Y* = 0.641. The AUC of the OPLS‐DA model used to predict the KRAS(+) mutation in tissues was 0.99. In Table S4, the prediction performance parameters, including accuracy, AUC, specificity, and sensitivity, determined by the OPLS‐DA model to classify KRAS genotype are provided.

The discriminatory molecules identified in KRAS(+) tissues are listed in Table 4. The selection criteria were as follows: VIP > 1, standard error of VIP < 1, and *p* < 0.05. All the molecules listed in Table 4 are different molecules from those listed in Table 1. Among the 24 identified as discriminatory molecules, four molecules were assigned as G‐PCs, phosphatidylcholine derivatives. As shown in Table 1, G‐PCs were identified as discriminatory molecules for the classification of CRC, but the mass values for the KRAS(+)‐related molecules were totally different from the mass values of these derivatives. By TOF‐SIMS analysis, several G‐PCs were newly identified as KRAS(+) discriminators.

**TABLE 4:** Discriminatory molecules for KRAS(+) mutation in tumor tissues analyzed by TOF‐SIMS analysis

Peak (*m*/*z*)	*p*‐value	VIP	Assignment	Formula
100.06	2.31 × 10^−4^	2.13	G‐PC	C_5_H_10_NO^+^
106.05	0.0182	2.28		
109.14	4.51 × 10^−4^	1.87	Cholesterol and derivatives	C_8_H_13_
112.06	1.60 × 10^−4^	1.81		
116.03	0.043	2.11		
119.05	0.036	1.54		
120.05	0.018	2.01		
121.04	6.15 × 10^−3^	1.77		
123.10	7.80 × 10^−4^	1.90		
125.09	2.30 × 10^−3^	1.86		
129.11	1.11 × 10^−4^	1.61		
130.09	1.15 × 10^−3^	1.61		
134.06	8.56 × 10^−3^	2.68	G‐PC	C_5_H_13_NPO_3_
136.05	2.07 × 10^−4^	1.34	G‐PC	C_5_H_15_NPO_4_
141.10	4.55 × 10^−5^	1.59		C_8_H_15_NO^+^
142.05	9.41 × 10^−3^	1.79	Phosphonosphingolipid	C_2_H_9_NPO_4_
144.08	1.61 × 10^−3^	1.51		C_10_H_10_N^+^
145.07	9.32 × 10^−3^	2.28		
152.06	3.60 × 10^−4^	1.88		
153.07	3.88 × 10^−4^	1.71		
155.08	5.87 × 10^−4^	1.91		
157.09	2.99 × 10^−3^	1.59		
159.09	2.45 × 10^−3^	1.74	Tryptophan	C_10_H_11_N_2_^+^
226.05	2.03 × 10^−4^	1.96	G‐PC	C_7_H_17_NPO_5_^+^

The mass value 159.09 was assigned to tryptophan,^29^ which was reported to be found in elevated quantities in KRAS‐mutated tissues.^43^ Tryptophan is catabolized to kynurenine, which is a tumor‐associated metabolite. Increased kynurenine levels have been observed in various tumors, and an important role of kynurenine is to suppress antitumor immune responses.^44^ In addition, the mass value 109.14 was assigned to cholesterol, which has also been reported to be elevated in tissues with a KRAS mutation.^45^ The steroid biosynthesis pathway was also significantly upregulated in KRAS(+) CRC cells, and the final product of steroid biosynthesis is cholesterol.^46^ KRAS(+) cancer cells require elevated levels of cholesterol to support their rapid growth.^47^

Altogether, the TOF‐SIMS spectra were able to classify the KRAS genotype in CRC tissues, and the several discriminatory molecules used for KRAS genotyping were well‐matched to the known indicators of KRAS mutations.

## 3 CONCLUSIONS

TOF‐SIMS is an imaging‐based mass spectrometric technique that can characterize the surfaces of biomaterials. Our study demonstrates the applicability of TOF‐SIMS as a metabolome screening method for cancer diagnosis. It allows the direct analysis of mass signals from tissue sections without requiring any extraction processes. However, TOF‐SIMS usually produces large amounts of raw data, which makes it difficult to extract valuable information using conventional software.

In this study, we applied a machine learning algorithm to manage TOF‐SIMS data from hundreds of patient samples. Preprocessing and optimization results for the SVM model for the classification of TOF‐SIMS data sets were provided in this report, and the data and method provided are potential computational resources for the utilization of metabolomic data in clinical decision‐making or biomedical research.

As we know, this is the first report confirming that KRAS genotyping can be achieved via mass spectrometric analysis. Based on these results, spectral data could be employed to determine treatment options for CRC patients. This work should also lead to improvements in our understanding of the involvement of lipids and metabolites in tumor progression and metastasis.

## 4 MATERIALS AND METHODS

### 4.1 Sample collection and preparation

Tissues from 90 CRC patients (45 male, 45 female) were obtained via the Keimyung University Dongsan Hospital Korea Regional Biobank and Inje University Paik Hospital Korea Regional Biobank, members of the National Biobank of Korea, which is supported by the Ministry of Health and Welfare. All samples derived from the National Biobank of Korea were obtained with informed consent in accordance with protocols approved by the review board at Daegu Gyeongbuk Institute of Science and Technology (IRB Approval No. DGIST‐150709‐BR‐015‐01). Normal and cancerous colon tissues were obtained from each patient (180 specimens). Tumor samples were provided with information on the percentage of tumorigenic mass in the whole sample. We used tumor samples with over 70% tumorigenic mass. For TOF‐SIMS analysis, ~1‐cm‐diameter specimens were frozen in optimal cutting temperature compound (Tissue‐Tek®) at −20°C. Cryosections of each tissue (10‐μm thick) were generated using a cryostat (Leica CM 1850, Leica Microsystems), placed on an indium tin oxide (ITO) glass slide and stored at −20°C. The slides were rinsed with water and then air‐dried immediately before analysis.

### 4.2 TOF‐SIMS measurement

Measurements were conducted on a TOF‐SIMS 5 instrument using a pulsed 30‐keV Bi_3_^+^ primary ion beam in spectrometry mode with a primary ion dose of 10^13^ ions/cm^2^ for positive ions. The primary beam irradiated the specimen at a 45° incident angle. Spectra were obtained from tissue sections over an area of 250 × 250 μm^2^ (256 × 256 pixels density). Three spectral points per a specimen were measured; thus, 540 mass spectra (270 normal and 270 tumor tissue spectra) were obtained. The mass range was *m*/*z* 100–1300 with a cycle time of 100 μs. The Bi_3_^+^ currents were typically 0.46 pA for spectrometry‐mode operation.

### 4.3 Optimization of the SVM model using TOF‐SIMS data to classify tumor tissues

The spectra from normal and tumor samples were analyzed using software written in the R language. Data preprocessing was performed for variance stabilization, normalization, spectrum alignment, averaging and peak detection, binning, and filtering. This preprocessing was performed using the MALDIquant package.^48^ Variance stabilization was performed by a square root transformation to overcome the potential dependency of the variance on the mean. For normalization, each intensity value was divided by the total ion intensities to overcome small batch effects. For the alignment of mass spectrometric data, a peak‐based warping algorithm was used. Subsequently, we averaged the spectral intensities to create a mean spectrum for each sample. Peaks were assigned to a local maximum above a noise threshold. Peak binning was performed by making similar mass values identical. Finally, less frequent peaks were removed by the filtering process.

After these preprocessing steps, PCA was performed using the mass spectra of each sample. The principal components for each sample were used as the input of the SVM classifier. ^21^ Cross‐validation was performed to tune the cost parameters in SVM. The overall data set was randomly divided into a training set (80% of overall set) and external testing set (20% of overall set) (Figure S2). The training set was then subdivided into three resample sets, and each resample set was also subdivided into analysis (80%) and assessment (20%) sets using random division. The analysis sets were used for SVM model development. Classification was then made for the associated assessment sets and then the external testing set. We evaluated the performance of the predictive model using sensitivity, specificity, accuracy, and AUC values.

### 4.4 Metabolomic analysis of CRC tissues using GC–TOF‐MS

A further twelve samples (six tumors and six normal tissues) from six patients were used for GC–TOF‐MS analysis. Each sample (100 mg) underwent a methanol extraction process; 1 ml of 100% methanol and 10 μl of internal standard (2‐chlorophenylalanine, 0.5 mg/ml) was used for the extraction in an MM400 mixer mill with a zirconium bead, and this was followed by sonication of the sample for 10 min. After incubation for 1 h at −20°C, cold centrifugation at 12,000 × g for 10 min was performed, and the supernatant was filtered through a 0.2‐μm polytetrafluorethylene filter and evaporated using a speed vacuum concentrator (Modulspin 31). The GC–TOF‐MS metabolomic analysis was performed according to the procedure reported by Yang et al. (2018).^49^ The GC system used in this study was Agilent 7890A (Agilent), and the mass spectrometer was Pegasus III. The column was Rtx‐5MS (30 m length × 0.25 mm × 0.25 μm). The gas flow rate was 1.5 ml/min, and the mass range was 50–1000 *m*/*z*. Metabolomic data were normalized using an internal standard.

The metabolomic data acquired by GC–TOF‐MS were classified by OPLS‐DA, using SIMCA P+ software (version 14.0). A score plot to visualize the classification was drawn, and the contribution of variables to the separation of classes was identified using the loading and contribution plots. The quality of the models is described by the cumulative modeled variation, via *R* ^2^ *X*(cum), *R* ^2^ *Y*(cum), and *Q* ^2^(cum) values. The performance of the prediction was evaluated by AUC and accuracy from the ROC curve. The VIP values according to their contribution to the model were identified, and VIP > 1 was used as a criterion for the discriminatory molecules. Significant differences between groups (*p* < 0.05), as calculated via *t*‐tests, were also considered.

### 4.5 Pathway analysis of potential biomarkers selected by the TOF‐SIMS and GC–TOF‐MS

For the selected CRC discriminators from TOF‐SIMS and GC–TOF‐MS measurements, related diseases and functions were analyzed to confirm their biological relevance as metabolite biomarkers. The identification of the discriminatory molecules was performed using literature studies and available online biochemical databases such as ChemSpider, Human Metabolome Database, and MassBank.jp (http://www.massbank.jp). The pathway analyses of the identified metabolites were performed using Ingenuity Pathway Analysis software (IPA) to identify enriched disease and bio‐function pathways due to the differential expression of the discriminatory molecules.

### 4.6 Classification of KRAS genotypes using TOF‐SIMS analysis

We tested 24 tumor samples for SNPs on KRAS G12D, G12A, and G12V sites (rs121913529). Before the pyrosequencing analysis of an SNP in the KRAS gene, a polymerase chain reaction (PCR) was performed using primer sets of forward 5′‐CGATACACGTCTGCAGTCAA‐3′ and reverse 5′‐ATCAAAGAATGGTCCTGCAC‐3′. Reactions were performed using 50 ng of genomic DNA, 10 μM of each primer, 2.5 mM of dNTP, and IP‐Taq DNA polymerase in a 25‐μl reaction volume. The PCR condition was 35 cycles of 30 s at 94°C, 30 s at 56°C, and 30 s at 72°C. The genotype of the PCR products was analyzed using a pyrosequencer. The DNA sequences were analyzed using the BioEdit program ver. 7.0.0 and the SNPs were identified by a manual search of the electropherogram results (data not shown). Using the OPLS‐DA model, we classified 72 TOF‐SIMS spectra for 24 samples according to the KRAS(+) versus KRAS(−) groups.

## CONFLICT OF INTERESTS

The authors have no conflicts of interest to declare.

### PEER REVIEW

The peer review history for this article is available at https://publons.com/publon/10.1002/btm2.10200.

## Supporting information


**Appendix S1:** Supplementary Information

## Data Availability

The data that support the findings of this study are available from the corresponding author upon reasonable request.
