# Supplementary material for: Screening of important metabolites and KRAS genotypes in colon cancer using secondary ion mass spectrometry
Source: Bioeng Transl Med. 2020 Nov 17;6(2):e10200. doi: 10.1002/btm2.10200 (PMC8126813; doi:10.1002/btm2.10200)
Supplement: Supplementary file 1 — Appendix S1: Supplementary Information [file BTM2-6-e10200-s001.pdf]

Supporting Information

Screening of important metabolites and KRAS genotypes in colon cancer using secondary ion mass spectrometry

Kookrae Cho,[1†] Eun-Sook Choi,[2†] Sung Young Lee,[3] Jung-Hee Kim,[1] Dae Won Moon,[4] Jong-Wuk Son,[1*] Eunjoo Kim[1*]

[1]Division of Electronic Information System Research, Daegu Gyeongbuk Institute of Science and Technology (DGIST), Daegu, Republic of Korea, 42988
[2]Division of Bio-Fusion Research, Daegu Gyeongbuk Institute of Science and Technology (DGIST), Daegu, Republic of Korea, 42988
[3]Division of Technology Business, National Institute for Nanomaterials Technology (NINT), Pohang University of Science and Technology (POSTECH), Pohang, Republic of Korea, 37673
[4]Department of New Biology, Daegu Gyeongbuk Institute of Science and Technology (DGIST), Daegu, Republic of Korea, 42988

† These authors contributed equally to this work.

*Corresponding authors:
Eunjoo Kim (ejkim@dgist.ac.kr)
Jong-Wuk Son (jwson@dgist.ac.kr)

Table S1. Preprocessing types and parameters for the SVM model using MALDIquant.

Type/Parameter	Setting
Variance stabilization	Square root method
Normalization	Total ion current method
Warping/Alignment	HalfWindowSize: 20<br>SNR: 2<br>Tolerance: 0.002<br>WarpingMethod: lowess
Peak detection	MAD method<br>HalfWindowSize: 20<br>SNR: 4–9
Peak binning	Tolerance: 0.002
Peak filtering	MinFrequent: 0.25

Table S2. Performance of SVM algorithm for the classification of normal vs. tumor tissues using TOF-SIMS measurement.

Type	SVM
Sensitivity	0.8387
Specificity	0.8817
Accuracy	0.8286
AUC	0.9207
Kappa	0.7204

Table S3. Performance of normal vs. tumor classification using GC-TOF-MS measurement.

	OPLS-DA
AUC	1.0
Accuracy	1.0
Sensitivity	1.0
Specificity	1.0
R2X	0.407
R2Y	0.878
Q2	0.431

Table S4. Performances of KRAS(+) vs. KRAS(-) classification models based on the evaluation parameters.

	OPLS-DA
AUC	0.9921
Accuracy	0.9048
Sensitivity	1.0
Specificity	0.7143
R2X	0.893
R2Y	0.641
Q2	0.399

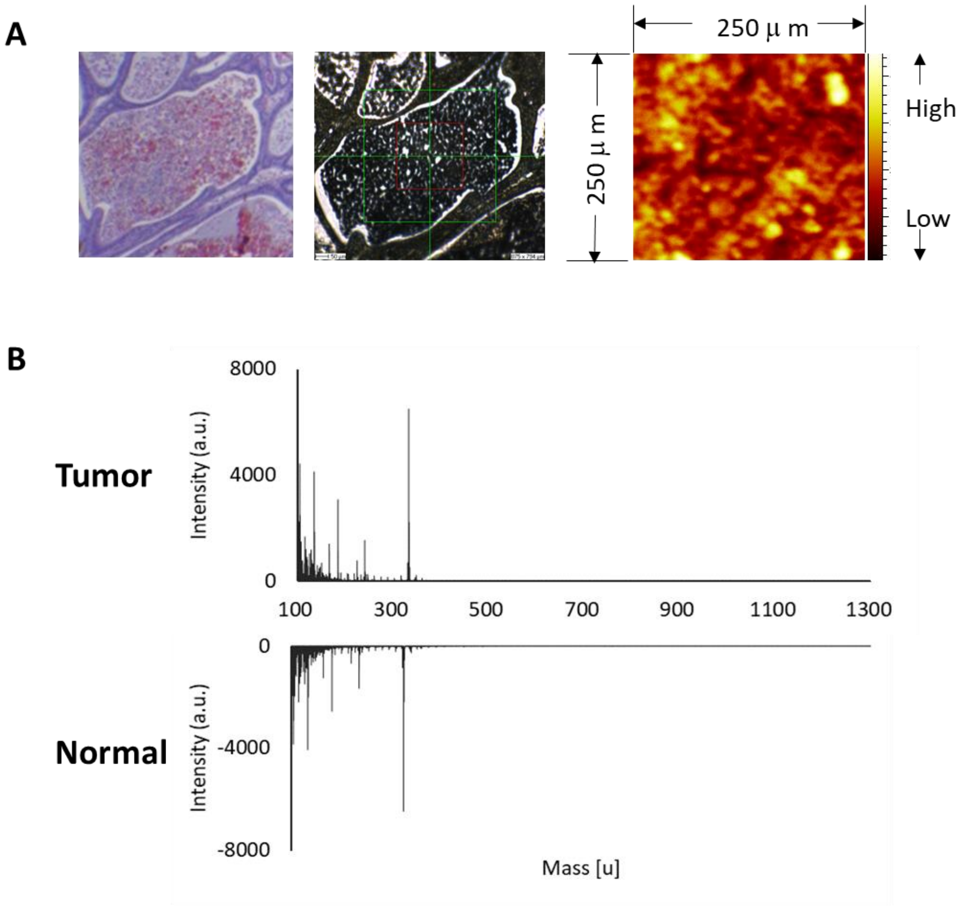

Figure S1. Representative images of TOF-SIMS analysis. (A) Tissue specimen analyzed by histological staining (right), optical image (middle), and image produced by total mass spectra (right)

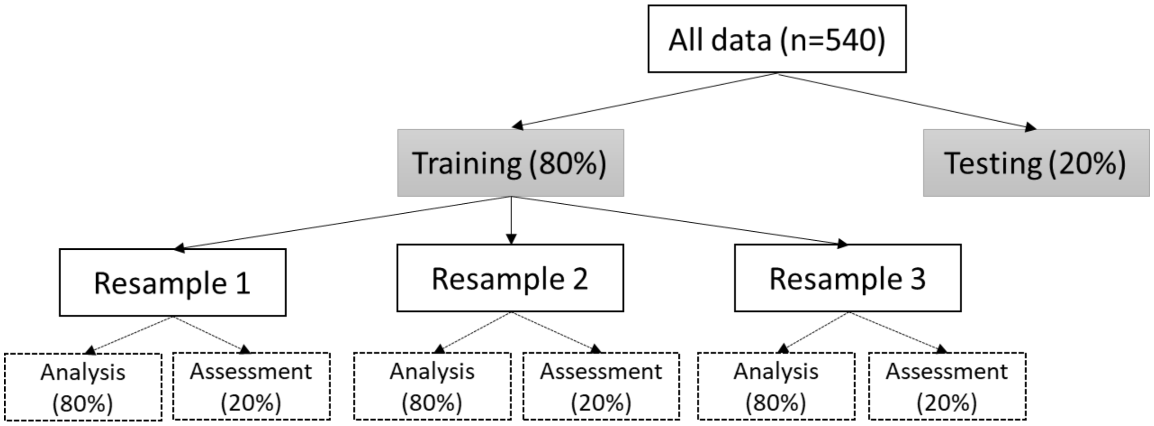

Figure S2. Schematic of cross-validation technique for the optimization of SVM model.
